# Epigallocatechin 3-Gallate Ameliorates Bile Duct Ligation Induced Liver Injury in Mice by Modulation of Mitochondrial Oxidative Stress and Inflammation

**DOI:** 10.1371/journal.pone.0126278

**Published:** 2015-05-08

**Authors:** Kezhen Shen, Xiaowen Feng, Rong Su, Haiyang Xie, Lin Zhou, Shusen Zheng

**Affiliations:** 1 Key Laboratory of Combined Multi-organ Transplantation, Ministry of Public Health, the First Affiliated Hospital, College of Medicine, Zhejiang University, Hangzhou, Zhejiang Province, China; 2 Collaborative Innovation Center for Diagnosis and Treatment of Infectious Diseases, the First Affiliated Hospital, College of Medicine, Zhejiang University, Hangzhou, Zhejiang Province, China; 3 Division of Hepatobiliary and Pancreatic Surgery, First Affiliated Hospital, Zhejiang University School of Medicine, Hangzhou, China; University of Navarra School of Medicine and Center for Applied Medical Research (CIMA), SPAIN

## Abstract

Cholestatic liver fibrosis was achieved by bile duct ligation (BDL) in mice. Liver injury associated with BDL for 15 days included significant reactive oxygen/nitrogen species generation, liver inflammation, cell death and fibrosis. Administration of Epigallocatechin 3-Gallate (EGCG) in animals reduced liver fibrosis involving parenchymal cells in BDL model. EGCG attenuated BDL-induced gene expression of pro-fibrotic markers (Collagen, Fibronectin, alpha 2 smooth muscle actin or SMA and connective tissue growth factor or CTGF), mitochondrial oxidative stress, cell death marker (DNA fragmentation and PARP activity), NFκB activity and pro-inflammatory cytokines (TNFα, MIP1α, IL1β, and MIP2). EGCG also improved BDL induced damages of mitochondrial electron transport chain complexes and antioxidant defense enzymes such as glutathione peroxidase and manganese superoxide dismutase. EGCG also attenuated hydrogen peroxide induced cell death in hepatocytes *in vitro* and alleviate stellate cells mediated fibrosis through TIMP1, SMA, Collagen 1 and Fibronectin *in vitro*. In conclusion, the reactive oxygen/nitrogen species generated from mitochondria plays critical pathogenetic role in the progression of liver inflammation and fibrosis and this study indicate that EGCG might be beneficial for reducing liver inflammation and fibrosis.

## Introduction

Metabolic liver disease, chronic alcohol drinking and viral hepatitis are major causative agents for chronic liver damage. Chronic liver damage leads to liver inflammation followed by fibrosis and cirrhosis, which is life threatening if not treated [1]. Chronic cholestatic liver disease with massive fibrosis is one of the most common occurrences after liver transplantation [2]. The most common treatment is ursodeoxycholic acid but it does not prevent fibrosis [3]. Antioxidants, inhibitors of cell death pathways and diet are tested against experimental animal models [4–7].

Epigallocatechin 3-Gallate (EGCG) is major component of green tea and has been used in traditional medicine in China [8, 9]. EGCG is a polyphenol and is a type of catechin. It is an ester of epigallocatechin and gallic acid The beneficial effect of EGCG has been reported in many liver disease model in animals such as ischemia/reperfusion injury, fatty liver, alcoholic liver damage and cancer [10–14]. Recently, clinical studies with EGCG dietary supplement demonstrated improved liver function in obese [15].

Liver fibrosis is manifested by significant deposition of extracellular matrix and stellate cells play a major role in the process [16]. Fibroblasts derive from hepatocytes also contribute to fibrosis [17].The specific role of myofibroblast and endothelial cells in developing liver fibrosis has also been reported [18, 19]. Thus, liver fibrosis is a complex process involving different cell types with specific role. The most important trigger for fibrosis is chronic inflammation and inflammatory cells plays a an orchestrated network with liver cell types to develop conducive environment for fibrosis [20].

Hepatocytes are most sensitive cells among liver cells when exposed toxic agents such as bile acids. The major cause of damage is mediated by ROS and leads to apoptosis. In liver fibrosis, damaged hepatocytes induce trigger signal by releasing cytokines which promote macrophage /kupffer cells and lymphocyte recruitment [21]. It is also proposed that fibroblast in fibrotic liver is mainly generated from those hepatocytes. In addition to that, hepatocytes also release paracrine molecules (such as fibroblast growth factor) which lead to stellate cell activation. Activated stellate cells with altered morphology secrete pro-inflammatory cytokines, induce adhesion molecules and generate extracellular matrix [22]. Activated HSC also converted to myofibroblastic phenotype which have contractile capability and differentiate into collagen producing cells and expressing myogenic and fibrotic markers such as smooth muscle actin, TGF –β etc.

In this study we investigated the role of EGCG in liver inflammation and fibrosis using *in vivo* mice model of bile duct ligation. We also provided evidence that EGCG selectively reduced mitochondrial injury by modulating oxidative damage and antioxidant defense. We also demonstrated that EGCG reduced hydrogen peroxide induced cell death in hepatocytes and attenuated production o fibrotic markers from stellate cells in vitro.

## Method

### Ethics Statement of the study

This study was performed under “Guide for the Care and Use of Laboratory Animals” of the National Institutes of Health. All protocols were approved by Animal Ethics Review Committees of Zhejiang University. All efforts were made to minimize suffering.

### Animal Experiments

Male inbred C57BL/6 (H2b) mice at 6–8 weeks old were purchased from the Animal Research Institution of Zhejiang Province (Hangzhou, China). Mice were housed under a standard SPF environment with a 12h dark-light cycle and free access to water and food. All animal experiments were conducted in accordance with the Guidelines for the Care and Use of Laboratory Animals and were approved by the Animal Ethics Review Committees of Zhejiang University.

### Bile duct ligation procedure

Bile duct ligation (BDL) surgeries were performed under anesthesia. The anesthesia was performed by intraperiitorial injection of xylazine at 10mg/kg and ketamine at 100mg/kg which allows mice to stay deep anesthetized condition for over 2 hours when the surgeries were carried out. The surgical area was shaved. The area was then be prepped by scrubbing the skin 3 times with povidone-iodine alternating with 70% ethanol. These survival surgeries involve a midline laparotomy (about 15mm) and bile duct was ligated with silk thread at two position as described earlier [4] and wounds were closed. In sham-operated control (refereed as control), the common bile duct was not ligated. Post-operative analgesic administration e.g. ketoprofen 5 mg/kg in sterile isotonic fluids, SQ, was given daily for at least 2 days after surgery The mice were sacrificed under 5% isoflurane anesthesia followed by cervical dislocation at 15 days after the surgery as described earlier [23, 24].

All mice were monitored every day for distress or pain. We have observed a death rate of 12% in 15 days BDL, which is in consistence with earlier report [25]. No adverse effect was observed. To test the effect of EGCG on BDL-induced hepatic fibrosis, mice received one dose of pre-operative (2 hour) IP injection of EGCG and alternate day postoperative IP injection of EGCG (30mg/kg/day) for two weeks. A total of 24 mice were performed with bile duct ligation where 12 of them treated with EGCG. For sham control and EGCG treatment we used 10 mice/group.

### Isolation of Mitochondria from fresh tissue

All procedure of liver tissues was performed at on ice to minimize mitochondrial-membrane and protein degradation. Mitochondria were isolated using commercial tissue mitochondria isolation Kit (Pierce, USA). Liver tissue from experimental animals were harvested and immersed in isotonic buffer provided with kit. Mitochondrial pellets were suspended in 500μl of RIPA buffer, and protein concentration was determined by using Bio Rad protein assay kit. The values obtained were corrected for BSA as standard.

### Mitochondrial protein 3-nitrotyrosine (3-NT) content

Hepatic 3-NT levels were determined with ELISA kit from Hycult biotechnology, Cell sciences, Canton, USA.

### Picro-Sirius Red staining

Quantitative determination of hepatic fibrosis was performed with Picro Sirius Red Stain kit (Abcam Company Ltd. China) and followed manufacturer’s instruction. The amount of red staining, marker for collagen staining was analyses from histological image using ImageJ software (NIH, USA). Images were analyzed from six random 100X fields from each animal and averaged.

### RNA isolation and Quantitative Real-time-PCR

RNA isolation was carried out Trizol reagents (Life Technologies, USA) according to previously published method [26]. Superscript III (Life Technologies, USA) was used for reverse transcription and Syber green (TaKaRa, China) method was employed for Real-time PCR. Real-time PCR were carried out in ABI 7500 instrument. Fold change was calculated as described earlier [27].The gene specific primers were purchased from Qiagen (USA).

### Mitochondrial complex I, II and IV activities

**A**ctivities of mitochondrial complex I, complex II, and complex IV were performed using Microplates assay kits according to the manufacturer's instructions. (Mitosciences, USA) Activities were calculated based on the manufacturer’s instruction on kinetic data and expressed as fold change.

### Quantification of MnSOD activity from mitochondrial fraction

MnSOD activity was determined from mitochondrial fraction using SOD activity kit (Enzo Life Sciences International, Inc., USA). SOD activity was determined from percent inhibition of the rate of WST-1-formazan formation with colorimetric readout at 450 nm. A kinetic assay was performed according to manufacturer’s instruction. SOD activity is expressed as fold change compared to sham control.

### Glutathione Peroxidase Assay from mitochondrial fraction

Mitochondrial glutathione peroxidase was measured using Glutathione Peroxidase Assay Kit (Abcam Company Ltd. China) according to manufacturer’s instruction. Glutathione peroxidase reduces cumene hydroperoxide while oxidizing GSH to GSSG. The generated GSSG is reduced to GSH with consumption of NADPH by GR. The decrease of NADPH (measured at 340 nm) is proportional to Glutathione Peroxidase activity. The activity was displayed as fold change compared to sham control.

### Western blot analysis

The protein content of of mitochondrial and cytosolic fractions was determined according to the Bradford method. Equal amounts of protein were loaded onto SDS-polyacrylamide gels and blotted onto nitrocellulose membranes. Western blots were performed by using antibodies directed against prohibitin (1:500, Abcam Company Ltd. China) and beta-actin (1:2000, Abcam Company Ltd. China), Secondary antibodies were purchased from FazendoMedia (Beijing, China).

### Hepatic PARP activity

PARP activities in the liver tissue were performed using commercial assay kit (Trevigen Inc.) according to instruction provided. The quantitative values were expressed as fold change compared to sham control.

### NFκB p65 binding ELISA

Nuclear lysates were assessed to quantify relative nuclear p65 transcript binding using kit from Abcam (China). In short, a specific double stranded DNA sequence containing the NFκB response element is immobilized onto the wells of a 96 well plate for ELISA analysis of p65 binding as detected by antibody binding and HRP conjugate detection. Absorbance was measured at 450nm. Both positive and non-specific binding controls were included.

### Primary hepatocyte isolation and culture

Hepatocytes from mice were isolated based on standard method as published earlier [28]. Hepatocytes were treated with one ROS component hydrogen peroxide (HP) for 24 hours at 5mM. EGCG was added at 10μM concentration. Cells were analyzed by flow cytometry for early apoptotic marker Annexin V APC and cell death dye Sytox green (Life Technologies, USA).

### Stellate Cells isolation and culture

Mouse stellate cells were isolated based on earlier published method [29]. Stellate cells were grown in 6 well culture plates for 8 days. EGCG were added at 10μM every day after adding fresh media. RNA was isolated from fresh (Day0) or after 8 days(Day8) and real-time PCR were performed as described before.

### Statistical Analysis

All data were presented as the means ± SEMs. Multiple comparisons (Tukeys) were performed using one way ANOVA using Graph Pad Prism software (USA). A p value less than 0.05 was considered statistically significant in all analyses.

## Results and Discussion

### EGCG attenuates BDL induced liver fibrosis in mice

**Fig 1:**
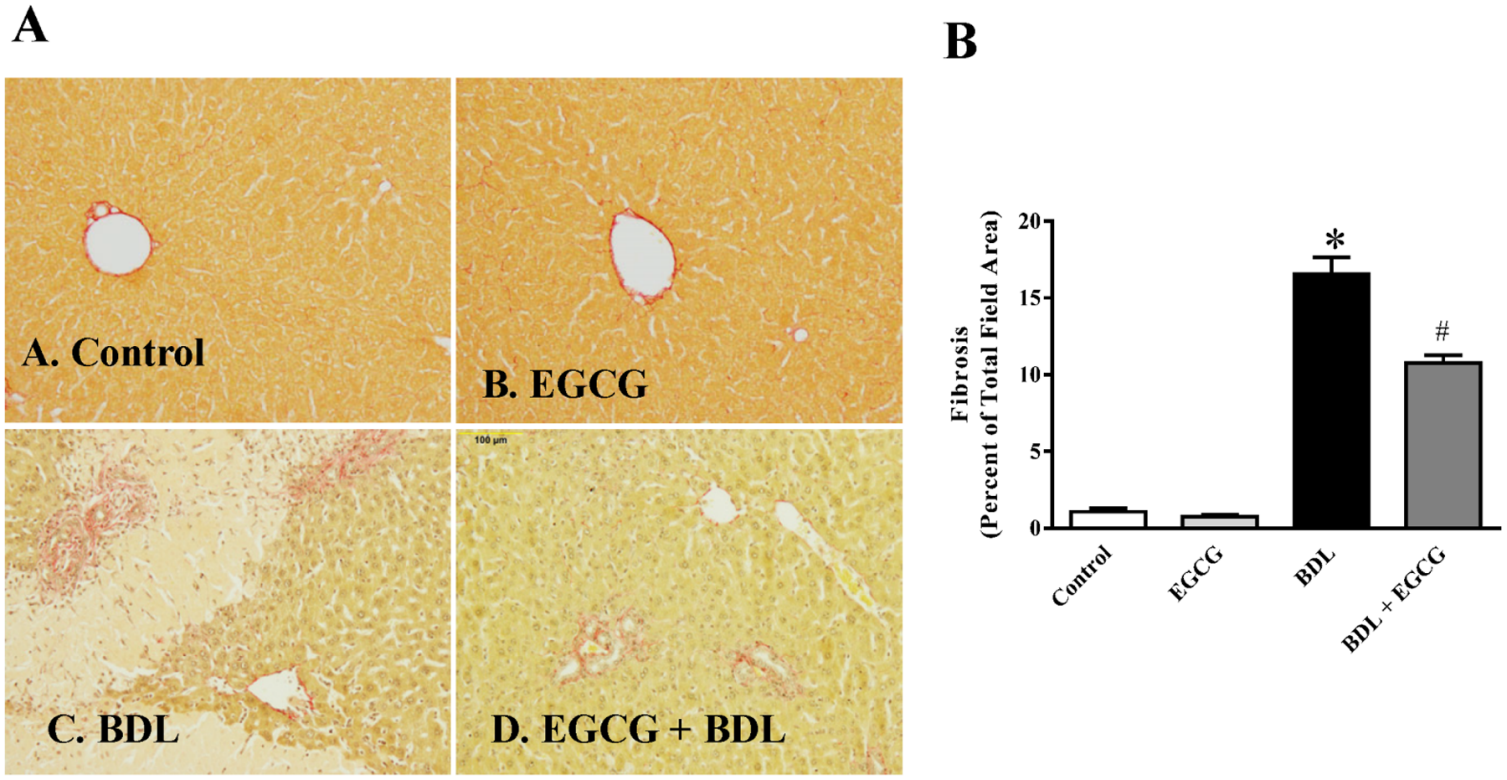
Effect of EGCG on BDL induced liver fibrosis in mice. BDL caused significant liver fibrosis as measured by Picrosirius Red staining (Panel A) and quantified by Image J (Panel B). BDL resulted in severe liver fibrosis which was attenuated by EGCG treatment. Results are mean ± S.E.M. n = 6/group.* p<0.05 versus control; and # p<0.05 versus BDL.

**Fig 2:**
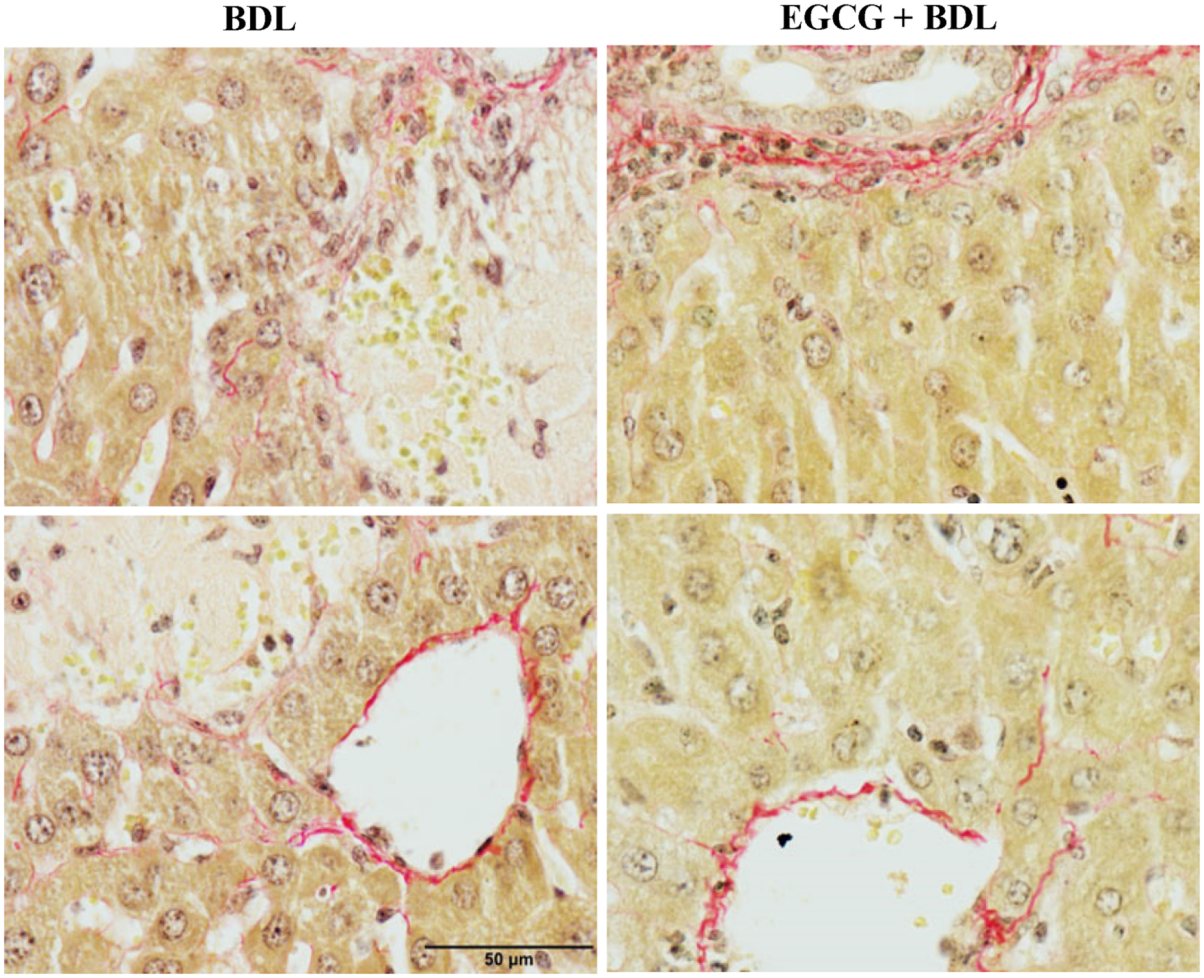
Effect of EGCG on BDL induced parenchymal cells. BDL caused significant liver fibrosis and necrosis to parenchymal cells as evident from 400X magnified images of histopathological staining. BDL resulted in severe fibrosis and necrosis which were attenuated by EGCG treatment.

**Fig 3:**
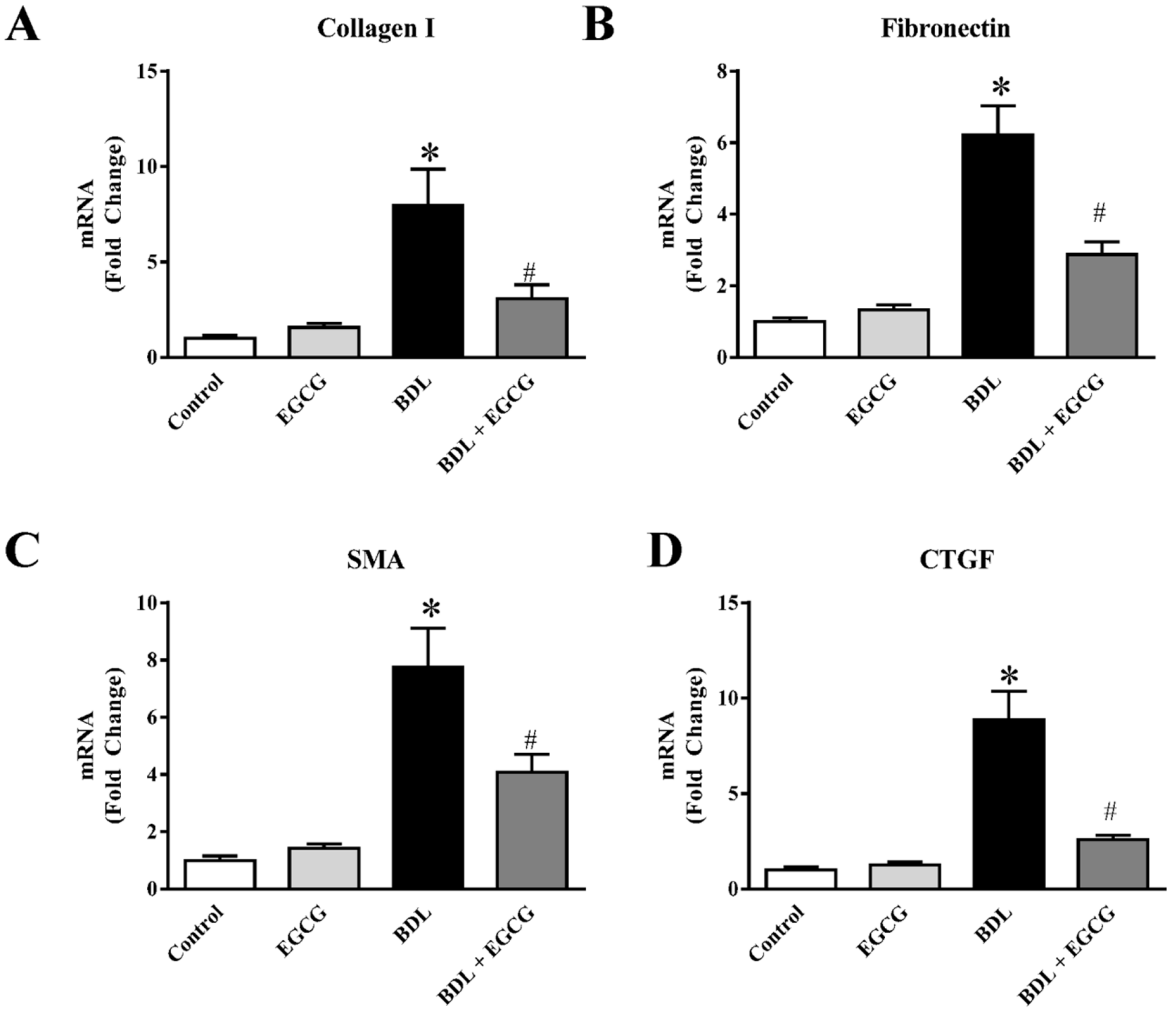
Effect of EGCG on BDL induced pro-fibrotic gene expression in mice liver. BDL caused significant increase in liver pro-fibrotic genes at mRNA level. Real-time PCR analysis of (A) collagen I, (B) Fibronectin, (C) alpha smooth muscle actin (SMA) and (D) CTGF. BDL induced increases in all four gene expression were attenuated by EGCG treatment. Results are mean ± S.E.M. n = 6/group.* p<0.05 versus control; and # p<0.05 versus BDL.

Chronic liver injury was achieved at 15 days after BDL procedure. BDL induced significant fibrosis as evident from picosirius red staining of collagen (Fig 1A). Treatment with EGCG on alternate day schedule at 30mg/kg dose reduced fibrosis. Quantitative measurement demonstrated that there is about 16 fold increase in fibrotic area due to BDL and EGCG reduced the fibrotic area up to 37% (Fig 1B). Further analyses of the images at higher magnification demonstrated fibrosis and necrosis of damaged parenchymal cells (Fig 2).We also examined expression of four key genes associated with fibrosis by real-time PCR All four genes namely collagen I, Fibronectin, SMA and CTGF were induced at mRNA level to 7.9 fold, 6.2 fold,7.7 fold and 8.8 fold respectively in BDL model as expected (Fig 3A–3D). Treatment with EGCG reduced BDL induced gene expression of collagen I, Fibronectin, SMA and CTGF to 62%, 54%, 48% and 70% of BDL level respectively. BDL is a well-established chronic liver injury model and is widely used [30]. BDL induces hepatocyte progenitor cells proliferation (such as biliary epithelial cells, oval cells) with associated portal inflammation and fibrosis [31, 32]. These processes lead to obstructive jaundice in two weeks and cirrhosis in 4 to 6 weeks [33]. The justification behind 15 days endpoint after BDL in this study was to investigate fibrosis as well as reduced mortality in mice was also considered [25]. This time point has been used earlier [4, 24]. The polyphenolic compound EGCG is the major catechin found in green tea and is thought to impart many of the health benefits [34]. The EGCG dose used for this animal study has been reported earlier and does not have any negative effect on liver or other vital organ [35–37]. In a recent human studies, bioavailability was between 1.6% at a low dose (75 mg/kg body weight) and 13.9% at higher doses (250 mg/kg and 400 mg/kg) when provided orally to healthy volunteers [38]. Protective effect of EGCG from BDL induced fibrosis demonstrated beneficial effect. EGCG shown to improve liver function in other liver injury model [10, 12–14] and reduced fibrosis in acute CCl_4_ liver injury [39].

### EGCG attenuates BDL induced mitochondrial oxidative stress by modulating mitochondrial antioxidant defense in mice

**Fig 4:**
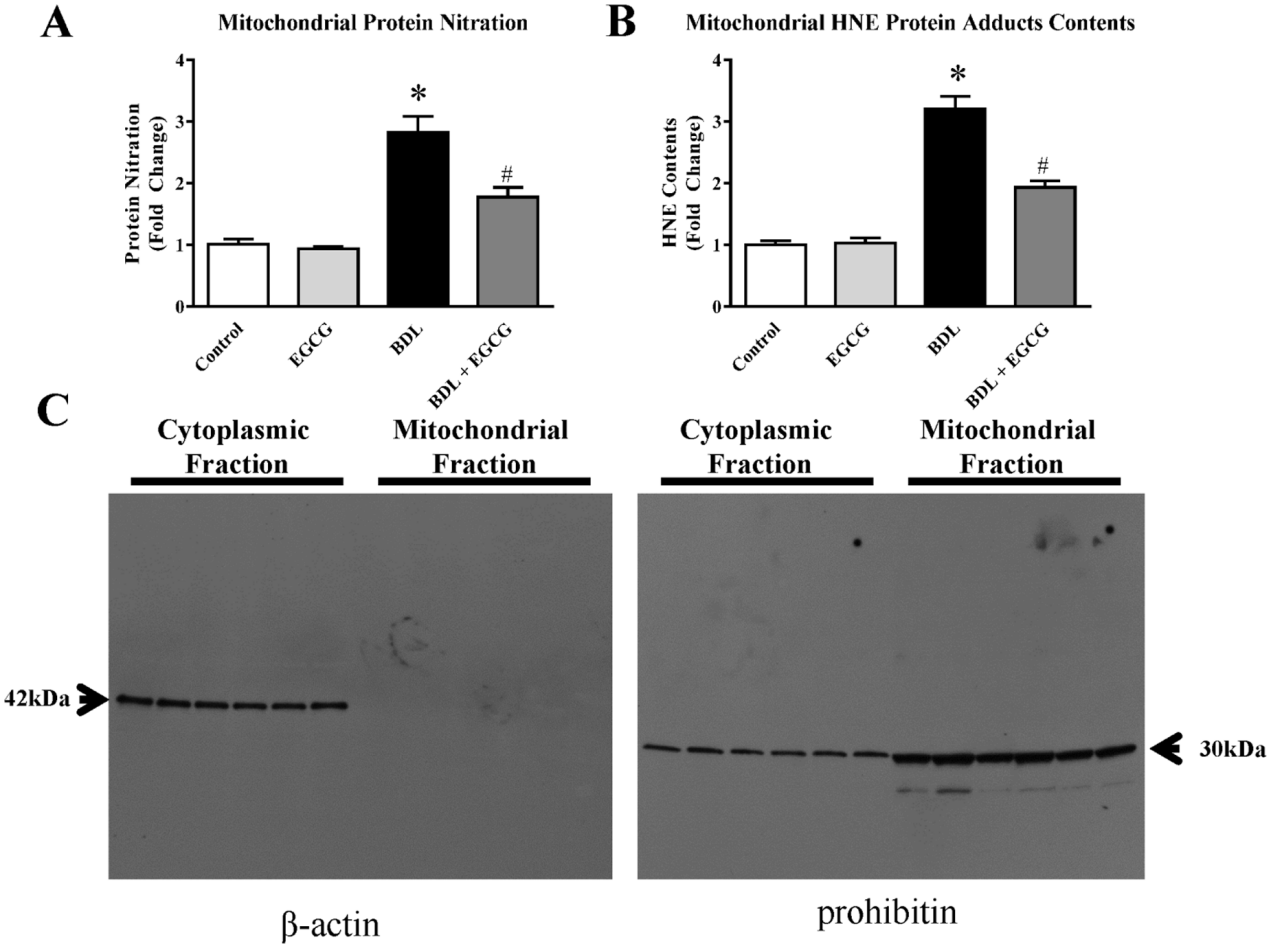
Effect of EGCG on BDL induced mitochondrial oxidative/nitrative stress in mice liver. BDL caused significant increase in mitochondrial oxidative and nitrative stress as measured by protein nitration and HNE protein adducts using commercial ELISA kits and these are footprints for nitrative and oxidative stress respectively. BDL induced nitrative stress (Panel A) and oxidative stresses (Panel B) in mitochondria were attenuated by EGCG treatment. Results are mean ± S.E.M. n = 6/group.* p<0.05 versus control; and # p<0.05 versus BDL. Western blot analyses for mitochondrial fraction and cytoplasmic fractions of same six samples for prohibitin (mitochondrial marker) and β-actin (cytoplasmic marker).

To understand the role of mitochondrial oxidative stress, mitochondria were isolated from liver and oxidative/nitrative markers were analyzed. HNE protein adducts from mitochondrial fraction increased significantly in liver due to BDL (3.2 fold) and EGCG treatment reduced significantly to 41% (Fig 4B). Mitochondrial protein nitration is also increased to 2.8 fold and EGCG attenuated 37% from BDL with vehicle group (Fig 4A). We also demonstrated the purity of mitochondrial fractions and separated cytosolic fraction from the same set of samples by western blot analyses using mitochondrial maker prohibitin and cytoplasmic market beta-actin (Fig 4C). BDL induced HNE or nitrated protein might be relevant for mitochondrial dysfunction. Oxidized or nitrated modified mitochondrial proteins significantly affect mitochondrial function [40–42]. Targets of protein nitration in mitochondria include key enzymes and electron transport chain complexes and this process is mediated by peroxynitrite [43, 44]. HNE modification of mitochondrial proteins has been reported earlier and is mediated by lipid peroxidation [45].

**Fig 5:**
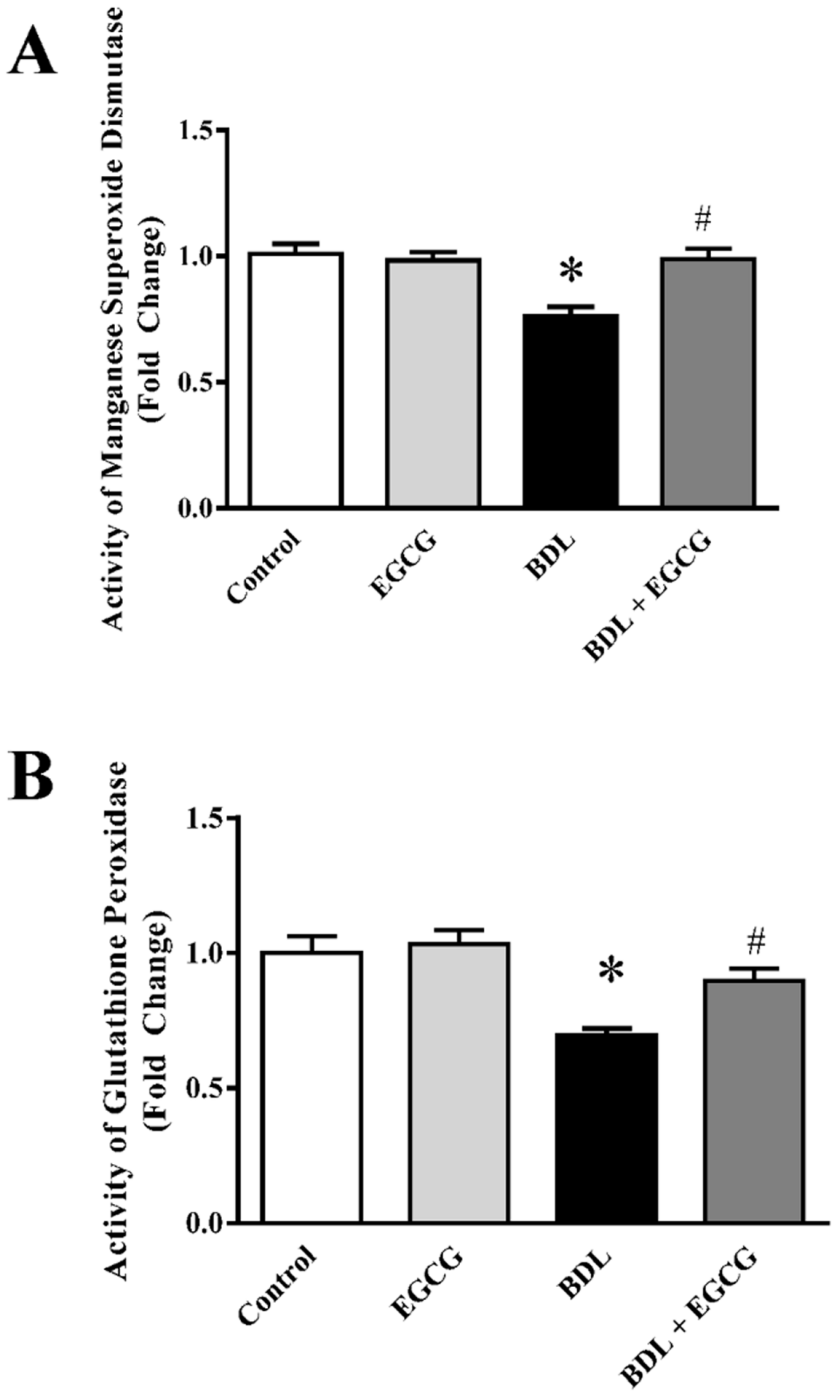
Effect of EGCG on BDL induced decrease of mitochondrial antioxidant defense in mice liver. BDL caused decrease in mitochondrial antioxidant defense as measured from enzyme activities of manganese superoxide dismutase (Panel A) and glutathione peroxidase (Panel B). BDL mediated drop in their activities in mitochondria were improved by EGCG treatment. Results are mean ± S.E.M. n = 6/group.* p<0.05 versus control; and # p<0.05 versus BDL.

Oxidative stress in mitochondria is modulated by its antioxidant defense mechanism. The balance of pro-oxidant and anti-oxidant in mitochondria is important for mitochondrial function in liver. We examined two anti-oxidant enzymes Glutathione Peroxidase and Manganese Superoxide Dismutase (Fig 5). BDL leads to 31% decrease in Glutathione Peroxidase activity in liver mitochondria and EGCG attenuated that reduced activity to control level. Activity of Manganese Superoxide Dismutase also reduced 24% compared to control group and restored to normal level by EGCG treatment. EGCG did not have any effect when administered alone (Fig 5). Loss of these enzyme activities may be contributed to oxidative modification, which was shown earlier [46–48]. The protective role of EGCG against mitochondrial oxidative stress might be due to its antioxidant capacity and it has been shown recently that EGCG can accumulate in mitochondria [49]. EGCG demonstrates mitochondrial protection in other tissue injury models [37, 50–54]. Many other similar plant polyphenol also have similar role in protecting against tissue injury [55–57].

### EGCG improves BDL impaired mitochondrial membrane complex activities in mice

**Fig 6:**
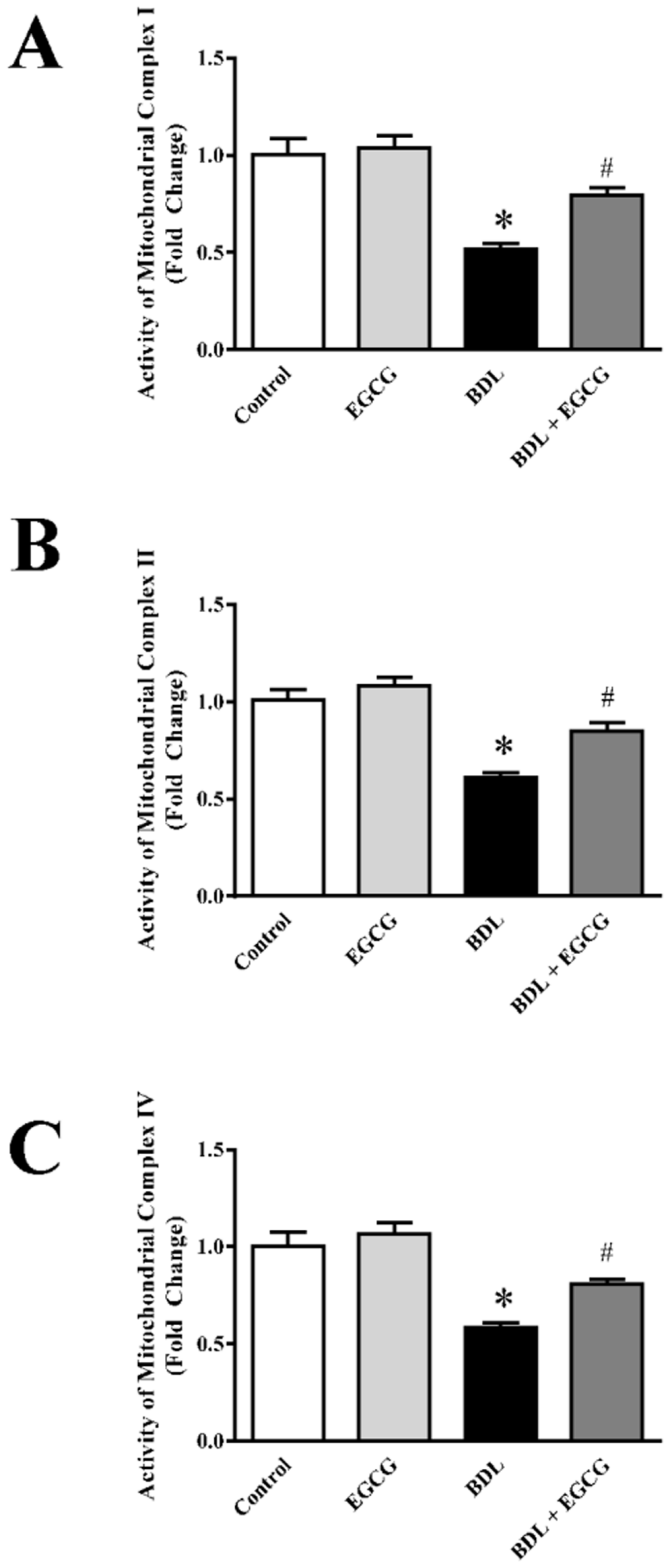
Effect of EGCG on BDL induced decrease of mitochondrial membrane complex activities in mice liver. BDL caused decrease in mitochondrial membrane complexes as measured from enzyme activities of electron transport chain complex I (Panel A), complex II (Panel B) and complex IV (Panel C). BDL mediated decrease of their activities in mitochondria were restored to control level by EGCG treatment. Results are mean ± S.E.M. n = 6/group.* p<0.05 versus control; and # p<0.05 versus BDL.

Mitochondria are the source of power in cells and electron transport chain is essential component for mitochondrial function. Here, we also examined electron transport chain complex activities from isolated mitochondria of liver. BDL procedure reduced complex I, complex II and complex IV activities to 49%, 39% and 42% respectively (Fig 6). Administration of EGCG during BDL increased complex I activity to 52%, complex II to 40% and complex IV to 38%. Thus EGCG administration significantly attenuated BDL induced mitochondrial dysfunction in liver. Electron transport chain complexes are also prone to oxidative modification and that leads its loss of activity [58, 59]. Selective accumulation of EGCG in mitochondria and its antioxidant properties is one of the mechanisms of protection from BDL injury in liver.

### EGCG attenuates BDL induced cell death pathway in mice liver

**Fig 7:**
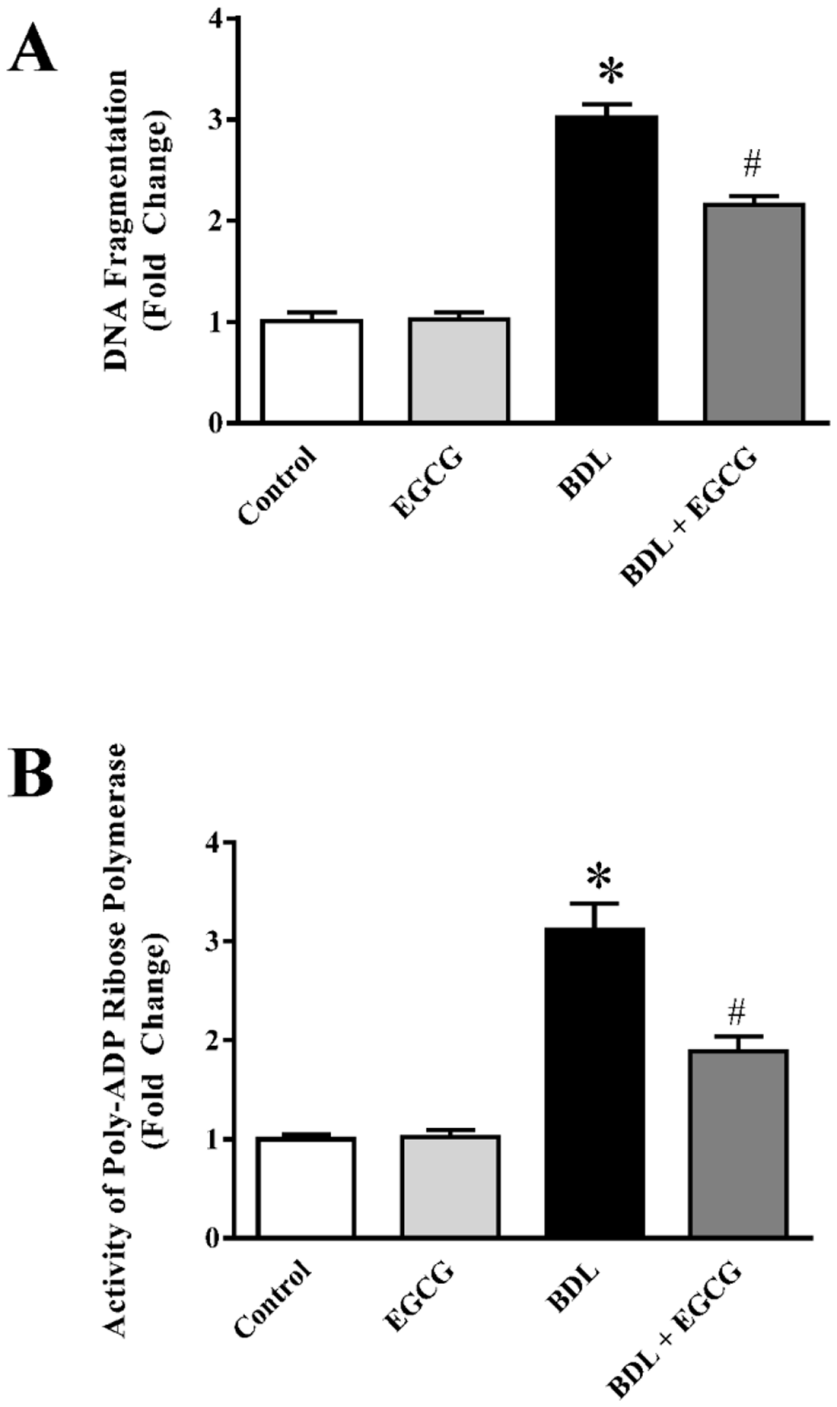
Effect of EGCG on BDL induced cell death markers DNA fragmentation and PARP activity in mice liver. BDL caused significant increase in liver DNA fragmentation (Panel A) and PARP activity (Panel B). BDL induced increases in DNA fragmentation and PARP activity were attenuated by EGCG treatment. Results are mean ± S.E.M. n = 6/group.* p<0.05 versus control; and # p<0.05 versus BDL.

BDL for two weeks induces significant cell death in liver [60, 61]. We also observed similar trend with cell death marker DNA fragmentation and PARP activity. BDL induced 3 fold increase in DNA fragmentation and EGCG attenuated 30% of BDL induced DNA fragmentation (Fig 7A). DNA fragmentation is predominantly apoptotic cell death marker. We also investigated another cell death marker PARP activity which is primarily known for necrotic pathway but also plays role in apoptotic pathway [62–64]. Chronic liver injury by BDL leads to 3.1 fold increase in PARP activity and EGCG administration attenuated BDL induced PARP activity to 40% (Fig 7B). In liver injury both apoptosis and necrosis play crucial role [65, 66]. PARP is a key mediator of liver fibrosis [4]. EGCG has been shown to be anti-apoptotic and it inhibits cell death [67, 68]. The major response to BDL induced cell death in liver is inflammatory trigger. The inflammatory response is mediated by neutrophil and other leucocyte accumulation [33, 69]. To understand the role of EGCG in BDL induced inflammation, we also looked the inflammatory cytokine and their master regulator NFκB.

### EGCG attenuates BDL induced NFB activation and pro-inflammatory cytokine

**Fig 8:**
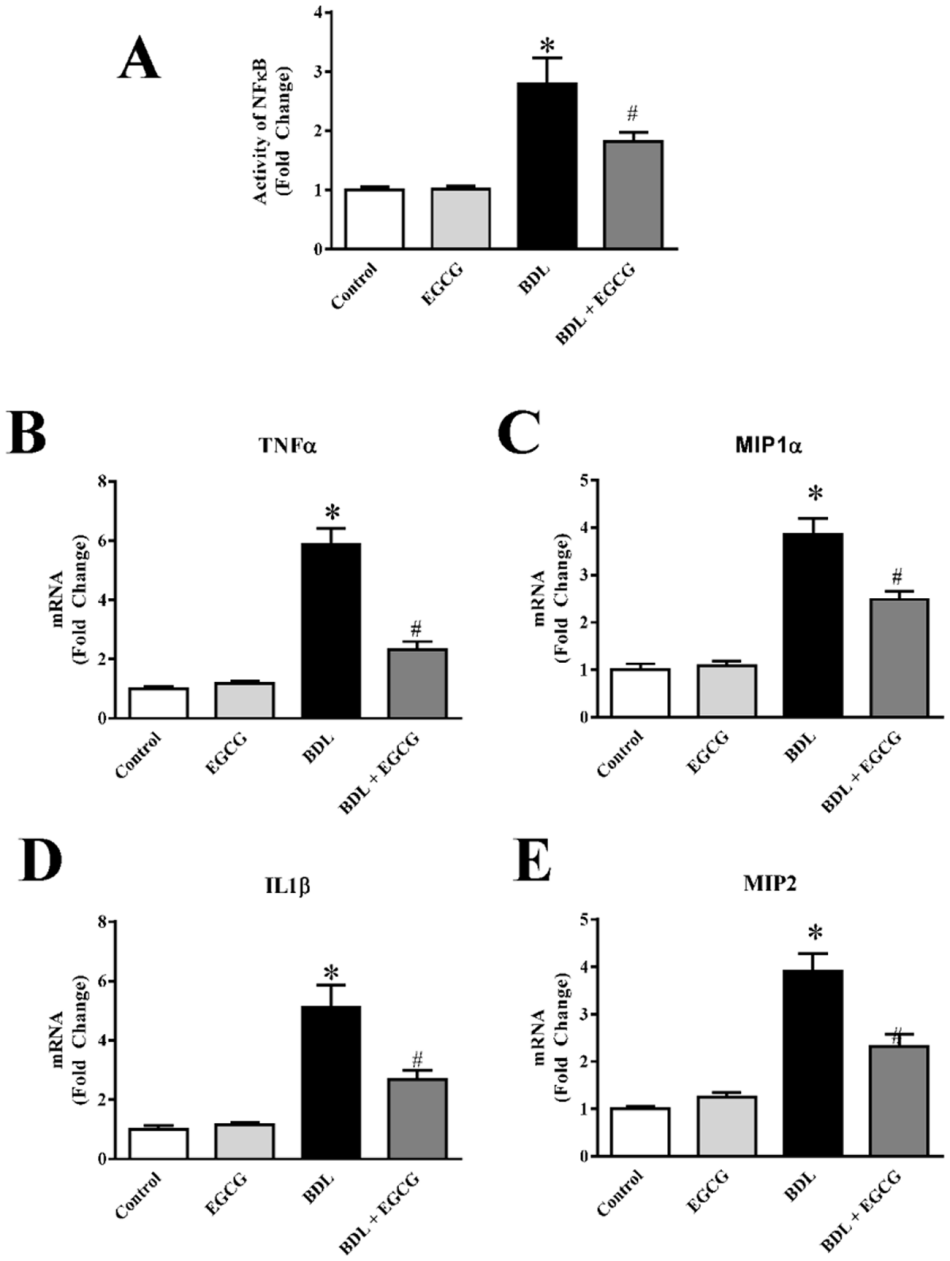
Effect of EGCG on BDL induced NFκB activation and pro-inflammatory cytokines in mice liver. BDL induced NFκB activation as measured from nuclear fraction using commercial kit (Panel A) and the activity was reduced by treatment with EGCG. BDL induced pro-inflammatory cytokines as measured by Real-time PCR analyses for TNFα (Panel B), MIP1α (Panel C), IL1β (Panel D) and MIP2 (Panel E). BDL induced increases in pro-inflammatory cytokines were attenuated by EGCG treatment. Results are mean ± S.E.M. n = 6/group.* p<0.05 versus control; and # p<0.05 versus BDL.

BDL induced fibrosis is mediated by inflammation and NFκB plays key role in these inflammatory diseases [70]. We have measured NFκB activity from nuclear fraction of liver homogenate. NFκB activity was increased (2.8 fold) in BDL group and the increased activity is reduced (upto 39%) by administration of EGCG during two weeks period (Fig 8). The modulation of NFκB activity by EGCG in cell line, immune cells and cardiac model has been reported earlier [71–74].

We also investigated pro-inflammatory cytokines TNFα, MIP1α, IL1β and MIP2. Real-time PCR analyses demonstrated BDL induced TNFα, MIP1α, IL1β and MIP2 mRNA level to 5.9 fold, 3.8 fold, 5.1 fold and 3.9 fold respectively (Fig 8B–8E). Administration of EGCG reduced BDL induced pro-inflammatory cytokines to 60.3%, 37%, 47.4% and 41% (TNFα, MIP1α, IL1β and MIP2 respectively). These pro-inflammatory cytokine are key mediator of inflammatory cell infiltration, associated oxidative burst and cell death. Recently EGCG demonstrate to modulate inflammatory response via PI3K/Akt/mTOR pathway [75].

### Mechanistic role of parenchymal cells in EGCG mediated anti-fibrotic action

**Fig 9:**
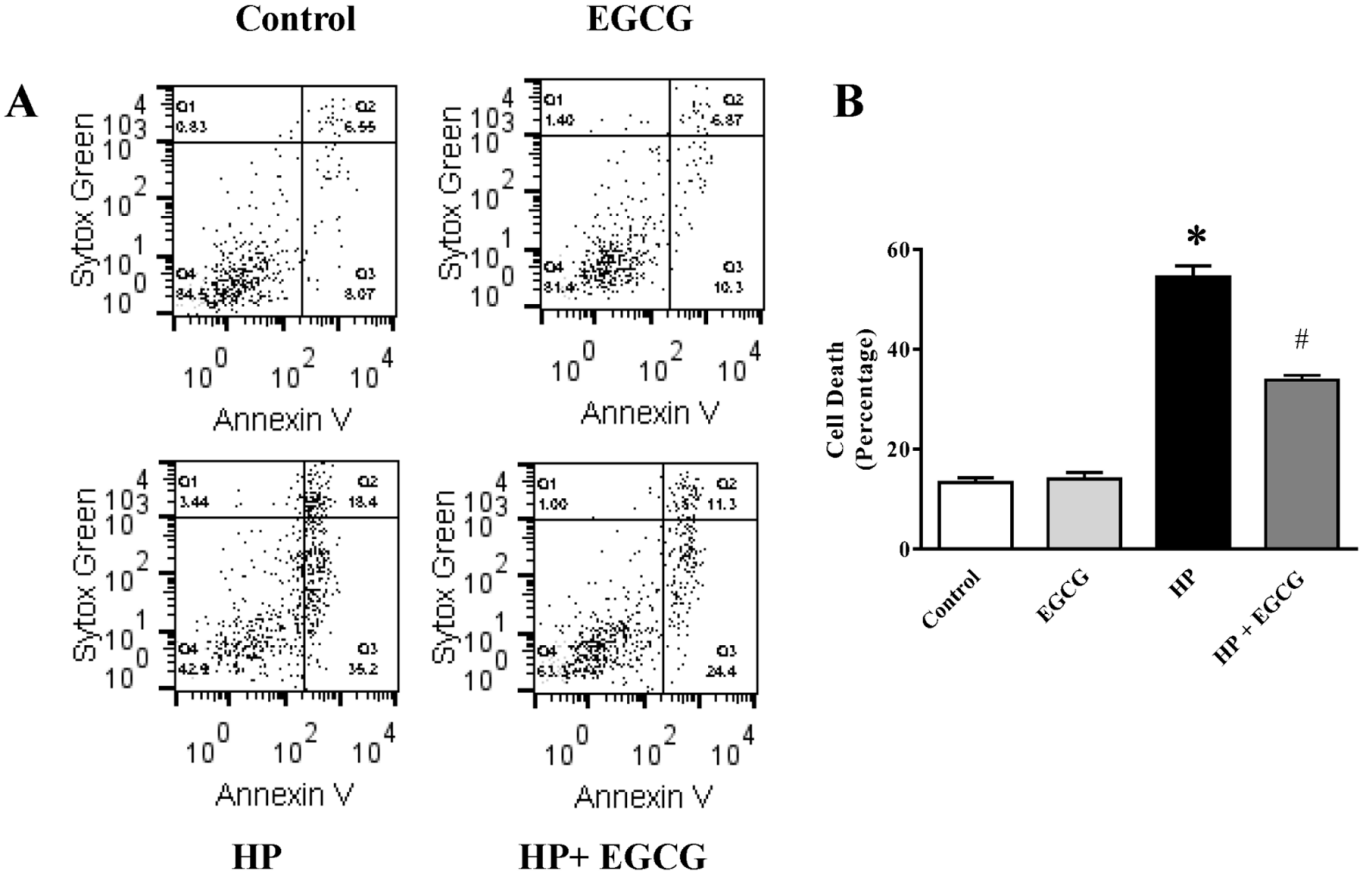
Effect of EGCG on ROS induced hepatocyte cell death. EGCG at 10μM inhibit hydrogen peroxide (HP, 5mM, 24h) induced cell death. Panel A. Representative dot blot analyses of flow cytometry in hepatocytes stained with early apoptotic marker Annexin V APC and cell death dye Sytox green. Panel B. Quantitative determination plot of cell death analyses (combination of early apoptotic and cell death) of the same experiments was presented. Results are mean ± S.E.M. n = 4/group.* p<0.05 versus control; and # p<0.05 versus HP.

To address the role parenchymal cells in EGCG mediated protection against fibrosis, we used isolated primary hepatocytes and stellate cells from mice and performed *in vitro* experiments. The initial response of accumulated bile acids is to generate significant oxidative stress and other toxic compounds, which damage sensitive hepatocytes. We want to understand the role of EGCG in reactive oxygen species induced hepatocyte cell death. EGCG treatment significantly reduced hydrogen peroxide(HP) induced hepatocyte cell death in vitro (Fig 9). It is well known that reactive oxygen species (ROS) exposure to hepatocytes followed by cell death plays critical role in liver fibrosis [48, 76]. EGCG significantly attenuated ROS mediated cell death and thus contributing anti-fibrotic properties.

**Fig 10:**
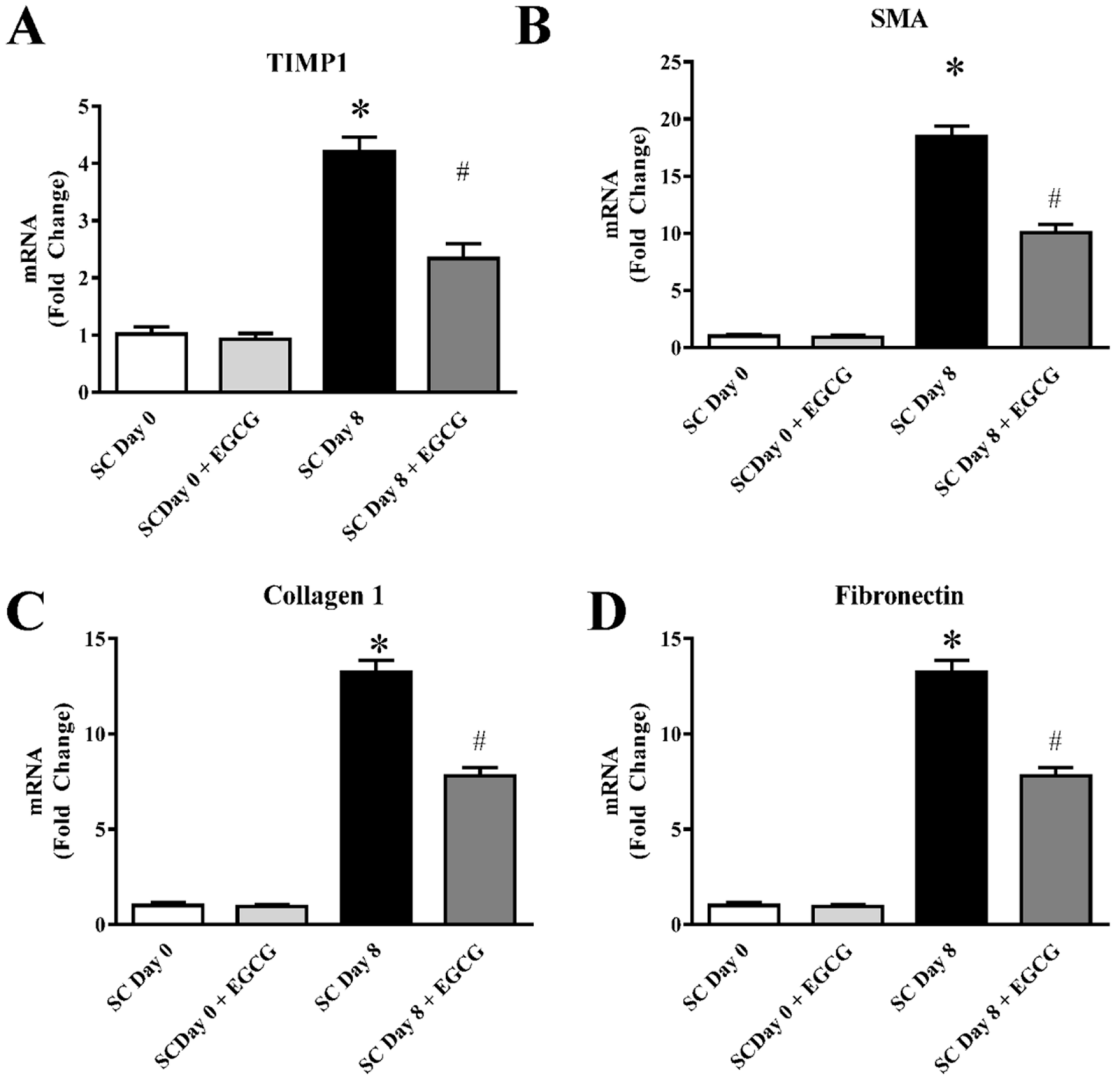
Effect of EGCG on pro-fibrotic gene expression in mouse stellate cells. Stellate were culture for 8days to stimulate fibrosis in culture and compared with fresh isolated stellate cells. Real-time PCR analyses of pro-fibrotic mRNA of TIMP1 (A), SMA (B), collagen1 (C) and Fibronectin (D) were induced during fibrosis stimulation. EGCG reverse the mRNA level in all four genes. Results are mean ± S.E.M. n = 4/group.* p<0.05 versus Day 0; and # p<0.05 versus Day 8.

In addition to hepatocytes, we also examine the effect of EGCG on stellate cell mediated pro-fibrotic gene expression. After receiving many paracrine signal molecules including cytokines, stellate cells activated and generate profibrotic molecules which leads to regulate the generation of extracellular matrix in mice liver. In primary culture of stellate cells, it was observed that stellate cells are activated spontaneously when cultures for 7 days or more [77]. Our aim is to understand the role of EGCG in the activation of stellate cell process and its effect on profibrotic marker production. We observed that TIMP-1, metallopeptidase inhibitor, is up regulated during collagen deposition in stellate cells (Fig 10A) However, treatment with EGCG significantly reduced upregulated TIMP1.TIMP-1, is a powerful inhibitor of enzymes that degrade matrix molecule and shown plays critical role in mouse fibrosis [78]. In liver fibrosis, it is important that upregulation of extracellular matrix protein (ECM) and matrix metalloproteinases (MMPs) mediated proteolytic degradation of ECM [79]. The rea-time PCR analyses demonstrated that EGCG treatment at 10 μM significantly reduced pro-fibrotic genes such as SMA, collagen1 and fibronectin (Fig 10B–10D). Stellate cells are one of the major player in progression of liver fibrosis [21].

Liber fibrosis is a complex process involving various parenchymal, non parenchymal and infiltrating immune cells. Protecting liver fibrosis or its reversal is a challenging task. Our data demonstrated that EGCG conferred significant protection and did not protect completely. This limitation of the study might be due to many factors including EGCG’s bioavailability, accumulation in liver and targeting other cell types. In addition to that multiple pathways are contributing factors including apoptotic pathway, renin angiotensin pathway, β-Catenin signaling, autophagy, angiogenesis, metabolic reprogramming and many others. It is difficult to understand that a single molecule EGCG can target all of pathways. However, hepatocyte cell death inhibition and preventing fibrotic marker from stellate cells by EGCG are very important contributions in protecting liver.

## Conclusion

**Fig 11:**
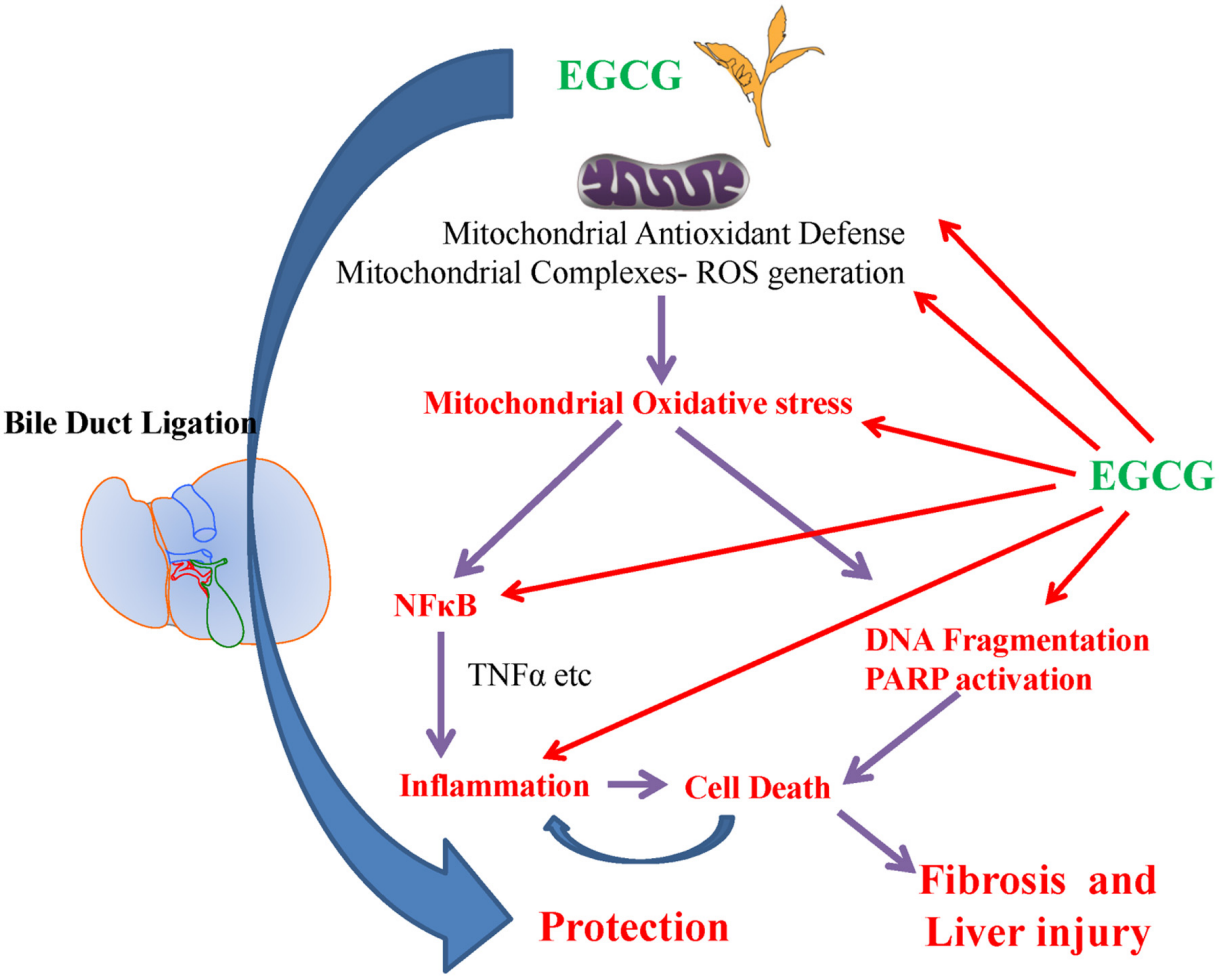
Schematic diagrams of protection mechanisms for EGCG in BDL induced livery injury. EGCG inhibit BDL induced mitochondrial ROS (Reactive Oxygen Species) generation which caused cell death. BDL induced cell death and NFκB activation leads to pro-inflammatory response with cytokines (TNFα, MIP1α, IL1β and MIP2). These process leads to leukocytes infiltration with additional burst of oxidative stress. EGCG also neutralize these pro-inflammatory cytokines. This multilevel interference by EGCG leads to reduced inflammation and cell death, thus protecting against BDL induced liver injury.

The basis of hepatic fibrogenesis is caused by pre-existing and renewed liver injury. The fibrogenesis is a complex process involving liver hepatocytes, stellate cells, kuppfer cells and infiltrating leukocytes. The current study shows that administration of EGCG was effective in attenuating BDL induced liver injury and fibrosis. The EGCG specifically attenuated of ROS induced hepatocyte cell death and down regulated pro-fibrotic gene expression in stellate cells. The protective effect of EGCG may be due to its ability to modulate mitochondrial oxidative/nitrative stress, NFκB activation and the inflammatory process (Fig 11). The detailed mechanism of action of EGCG in BDL induced liver fibrosis will require future studies with mitochondrial accumulation of EGCG and its mechanism of NFκB regulation will allow us to better understand the effects of EGCG on liver fibrogenesis.
